# Systemic manifestations of hepatitis C infection

**DOI:** 10.1186/s13027-016-0076-7

**Published:** 2016-05-23

**Authors:** Lydia Tang, Lauren Marcell, Shyam Kottilil

**Affiliations:** Division of Clinical Care and Research, Institute of Human Virology, University of Maryland School of Medicine, 725 W Lombard St, Room S222, Baltimore, MD USA

**Keywords:** HCV, Cryoglobulinemia, Autoantibodies, Lymphoma

## Abstract

Chronic hepatitis C (HCV) is a common infection affecting 185 million people worldwide. The most common manifestation of chronic HCV is progressive liver fibrosis, cirrhosis, liver failure and hepatocellular carcinoma. However, several systemic manifestations of HCV have been recognized and reported in the literature. The purpose of this review is to assimilate published literature based on evidence to categorize these extrahepatic manifestations with the likelihood of a causal association with HCV. Exciting recent developments have resulted in simple all oral interferon-free highly effective therapy for HCV. However, this treatment is also expensive and less accessible to most affected individuals as treatment recommendations are based on stage of liver fibrosis. Expanding the scope of HCV therapy to those with extrahepatic manifestations beyond what is currently recommended will significantly reduce the morbidity and mortality in this aging population.

## Background

Hepatitis C (HCV) is a single stranded RNA virus belonging to the family *Flaviviridae* and genus Hepacivirus [1]. It preferentially infects hepatocytes and establishes a chronic infection in approximately 85 % of patients with acute infection [2]. Globally, over 185 million people are affected by chronic HCV [2]. Chronic HCV infection can lead to cirrhosis of the liver, liver failure and hepatocellular carcinoma, and is the most common cause of liver transplantation in the USA and Western Europe [2]. Highly effective, interferon-free, direct acting antiviral (DAA) agents for chronic HCV are now available, resulting in sustained virologic response ([SVR], considered functional cure), for the majority of patients able to access the medications. The high cost of DAA therapy has resulted in prioritization of patients for therapy. Prioritization is most frequently based on the stage of liver disease; however, chronic HCV is actually a systemic disease and is associated with several debilitating extrahepatic manifestations that should be taken into consideration for prioritization of HCV therapy. To date, there has not been any correlation between the extent of systemic symptoms and stage of liver disease. As a result, these manifestations are often overlooked by providers when deciding who should benefit from the expensive DAA therapy. This review focuses on categorizing the clinical syndromes reportedly associated with chronic HCV based on the strength of epidemiological evidence. This approach will allow clinicians to prioritize therapy for patients with extrahepatic symptoms.

### Overview of HCV as a systemic disease

HCV is a hepatotropic virus; however, the chronic HCV infection can result in global health impairment, with up to 74 % of patients experiencing some form of extrahepatic manifestation [3]. These can be present long before the stage of advanced liver disease and include non-specific symptoms such as fatigue, nausea, abdominal or musculoskeletal pain, loss of weight, and neuropsychiatric symptoms including depression, irritability and malaise; and more specific biological manifestations such as cryoglobulinaemia vasculitis, lymphoproliferative disorders such as B-cell non-Hodgkin lymphoma, renal disease, type II diabetes mellitus, cerebrovascular and cardiovascular events, porphyria cutanea tarda and lichen planus. Table 1 summarizes the manifestation that will be reviewed in this review, organized according to the strengths of association with HCV. The importance of extrahepatic disease has been highlighted in a large prospective natural history study from Taiwan. In this study, 1095 patients positive for HCV antibody (AB) were followed for a mean duration of 16 years and HCV AB seropositivity was associated with increased non-liver-related mortality: cumulative 18-year mortality due to extrahepatic disease was 19.8 % compared to 11.0 and 12.2 % among those who spontaneously cleared their HCV infection and those who never acquired HCV, respectively. Multivariate–adjusted hazard ratio for non-liver causes of death was 1.47 (95 % CI, 1.23–1.77) among those who were HCV RNA positive, and chronic HCV infection was associated with an increased (1.4-fold) mortality from circulatory diseases [4]. Taken together, strong epidemiological data support that HCV as a systemic disease, associated with significant extrahepatic morbidity.

**Table 1:** A summary of the extrahepatic Manifestations of Hepatitis C discussed in this review

Extrahepatic manifestations
Most likely Associated with HCV
Mixed Cryoglobulinemic Syndrome
B-Cell Non-Hodgkins Lymphoma
Probably Associated with HCV
Diabetes Mellitus Type 2
Membrane Proliferative Glomerulonephritis
Neurological Impairments
Sicca Syndrome
Porphyria Cutanea Tarda
Lichen Planus
Possibly Associated with HCV
Autoantibodies
Moorens Corneal Ulcers
Cardiovascular Events

### The systemic benefits of HCV cure

Achievement of SVR has been shown to result in reduction in liver related and all cause mortality in several recent studies. The non-liver related mortality was significantly reduced in a large European cohort followed for over 7 years after achieving SVR [5]. In the United States, a large study conducted at the Veterans’ Administration also demonstrated lower non-liver related mortality attributable to HCV comorbidities such as diabetes, hypertension and cardiovascular disease [6]. A third, multi-center study that followed patients with SVR over 8.4 years also demonstrated a significant reduction in liver-related as well as all cause mortality [7]. More recently, Hsu et al. analyzed the effect of HCV treatment on adverse outcomes (death, end stage renal disease, ischemic stroke, and acute coronary syndrome) among patients with HCV. The incidence of these events were significantly lower among patients treated for HCV [8], a benefit that was also demonstrated among patients with co-existing diabetes mellitus [9]. These patients were treated with interferon-based therapy. The impact of treatment on other extrahepatic manifestations has not been as clearly demonstrated. We anticipate that DAA therapy, with its improved efficacy and anticipated wide ranging uptake, will allow the benefits of SVR to be more readily characterized. Awareness of clinicians to seek out and treat patients with HCV comorbidities is key in achieving this goal.

The remainder of the review will focus on non-hepatic diseases associated with chronic HCV infection. Table 1 summarizes the diseases reviewed. We will summarize the evidence supporting their association with HCV, including, if available, evidence of amelioration with HCV clearance.

#### Manifestations most likely associated with chronic HCV

These are associated conditions that have the strongest clinical prevalence data with chronic HCV [10, 11]. These include mixed cryglobulinemic syndrome (MCS) and B cell non-Hodgkin’s lymphoma.

##### Mixed cryoglobulinemic syndrome

MCS is a small to medium vessel vasculitis characterized by the expansion of B cells resulting in production of cryoglobulins: immune complexes of polyclonal immunoglobulin (Ig) G and monoclonal or polyclonal IgM with rheumatoid factor that precipitate at lower temperatures [12]. These fix complement and lead to endothelial tissue damage and vasculitis [10, 11]. The resulting syndrome can manifest in skin, major joints, peripheral nerves, and renal disease. As high as 90 % of cases of MCS are associated with chronic HCV [13]. Dermatological manifestations are the most common with palpable purpura [10, 11]. Other manifestations include arthritis, non-healing ulcers, peripheral neuropathy, central nervous involvement, and glomerulonephritis [10, 11]. The diagnosis is confirmed by the presence of cryoglobulins, elevated rheumatoid factor and immunofluorescence of complement fixing IgM in tissues.

Treatment of HCV-associate MCS may target HCV, or the downstream B cell arm of autoimmunity. Until recently, HCV therapy was dependent upon interferon and mixed cryoglobulinemia is identified as a negative prognostic factor of virologic response [14]. In a large prospective study of HCV treatment with pegylated interferon alpha among 253 patients with MCS or mixed cryoglobulinemia (circulating cryoglobulins but without MCS), 48.6 % achieved SVR. All but 2 of the 121 patients with MCS saw clinical-immunological resolution at 6 month follow up. Among the patients with MCS who did not achieve SVR, a transient improvement the syndrome was seen in some patients by the end of treatment; however, improvements did not persist at follow up [14]. This study supports that of an earlier, smaller study where HCV viral relapse occurred within 2 months of follow up in 8 patients, 6 of whom also saw a relapse in MCS [15]. The first generation NS3/4A protease inhibitors boceprevir and telaprevir were among the earliest DAA agents to be approved for HCV therapy. Triple therapy with pegylated interferon, ribavirin, and telaprevir or boceprevir was studied among participants with MCS in a pilot study. In this study, SVR rates were still low despite addition of boceprevir with none of the 5 patients with MCS and only 5 out of 16 of patients with mixed cryoglobulinemia without symptoms achieving SVR [16]. In contrast, a more recent study of 30 patients with MCS reported improved SVR (66.7 %) rates, with 70 % or patients achieving complete resolution of MCS clinical parameter [17]. This regimen, however, was associated with significant side effects. Newer DAA agents have excellent tolerability, are highly effective when used in combination, and do not require concurrent interferon therapy for the vast majority of patients. Subsequently, triple therapy with the first generation protease inhibitors is no longer recommended for HCV therapy. There are few publications reporting the efficacy of novel DAA therapy among patients with MCS. A case report documents successful clinical response associated with SVR in one patient with severe MCS after 12 weeks of combination therapy with ombitasvir, paritaprevir, ritonavir, and dasabuvir [18]. The VASCUVALDIC study was a prospective study that treated 24 patients with MCS with sofosbuvir and ribavirin. By end of treatment 87.5 % of patients had complete clinical response (improvement of all affected organs and absence of clinical relapse). Cryoglobulin disappearance occurred in 46.1 % of cases, and overall SVR was 74 %. Gragnani et al. treated 17 (7 with mixed cryoglobulinemia, and 10 with MCS) patients with interferon-free, DAA-based therapy and report improvement in clinical parameters in all the patients with MCS [19]. Sise et al. treated 12 patients with HCV-associated MCS (including 7 with renal involvement) with sofosbuvir (an NS5A inhibitor and the backbone of most DAA-based therapies currently approved for use in USA and Europe) in combination with either simeprevir (an NS3A/4A protease inhibitor) or ribavirin. The majority of patients were treated with 12 weeks of therapy and overall SVR rate was 87 %. Six of the patients with renal involvement achieved SVR, and this was associated with improvement in renal function and reduction in proteinuria. Cryoglobulin levels at follow up decreased for all patients compared to baseline, except for one patient who experienced virological relapse, with complete cryoglobulin loss in 4 of the 9 patients with levels available at follow up [20]. Importantly, 5 patients were on immunosuppression (4 with rituximab, and1 with ustekinemab for psoriasis) while on HCV treatment and several were able to reduce immunosuppression with only 1 (who experienced virologic failure) requiring increase in immunosuppression [20].

Several studies have supported the efficacy and safety of rituximab—a monoclonal antibody against CD20 that results in B cell depletion—for the treatment of HCV-associated MCS [21]. Only 2 prospective, randomized controlled trials have been published so far, reporting success in attaining remission and symptomatic relief [22] and greater survival (64.3 % versus 3.5 %, *P* < 0.0001) at month 12 [23] among patients who did not achieve SVR with antiviral therapy or intolerant to interferon alpha and ribavirin. A systemic review by Visentini et al. found that 32 % of patients experienced relapse after clinical response after rituximab monotherapy [21]. The same authors conducted a phase II study of low-dose rituximab for the treatment of MCS and reported a 41 % relapse [21]. Therefore, the proportion of patients who relapse after clinical response with rituximab monotherapy remains sizeable, solidifying the need for virological treatment.

MCS is a multisystem disease with significant morbidity. Current evidence supports the resolution of MCS for most patients who are treated for HCV and achieve SVR. Therefore, patients with mixed cryoglobulinemic vasculitis should be prioritized for treatment with current DAA-based therapy. It is expected that over time, this syndrome will be occurring with lower frequency.

##### B cell Non-Hodgkin’s Lymphoma (NHL)

HCV is also a lymphotropic virus and since 1994, B cell NHL has been associated with chronic HCV infection [24]. It is conceivable that B cell NHL is a progression of B cell expansion observed in mixed cryoglobulinemia as this condition is often described as a precursor of NHL [25]. These patients are at much higher risk (about 35 times more than the normal population) of developing NHL [26]. Chronic antigenic stimulation of B cells result in a monoclonal expansion of B cells (possibly mediated by CD81 engagement) gaining oncogenic potential over time [27]. A meta analysis of 15 studies suggested in countries where HCV AB seropositivity is high, about one-tenth of all NHL are attributable to chronic HCV infection [28]. A retrospective study comparing patients with HCV and not treated with patients who received interferon therapy found that the cumulative rates of malignant lymphoma (including diffuse large cell and follicular lymphoma) was 0.62 % at the end of the 5^th^ year, 2.26 % at the 10^th^ year, and 2.62 % at the 15^th^ year. This did not differ among those who did not respond to interferon therapy. Importantly, among patients who were successfully treated of HCV, cumulative rate of lymphoma was 0 % at all timepoints up to 15 years [29]. This is further supported by observations of remission of NHL and a positive impact on survival with HCV treatment of HCV [30–33]. Literature reporting the outcome of HCV treatment with interferon-free, novel DAA-based therapy is currently still limited to case reports. These report regression of lymphoma with successful HCV treatment [34–37]. As DAA-based HCV therapy becomes more widely used, the results from larger cohorts will hopefully be reported.

#### Manifestations likely associated with chronic HCV

These are clinical symptoms and disease manifestations that occur at higher frequency in chronic HCV than general population, although clinical association is not supported by strong epidemiological data.

##### Diabetes mellitus type 2

Several studies have demonstrated that chronic HCV is associated with a high prevalence of insulin resistance [38, 39] and diabetes mellitus [38, 40]. Patients with chronic HCV have a 2.3 fold increased chance of having type 2 diabetes mellitus [41]. HCV-infected Huh 7.5 cells expressed higher levels of peroxisome proliferator-activated receptor-gamma coactivator alfa (PGC1-alfa) and glucose production [42]. Diabetes mellitus has also been shown to alter the course of chronic HCV infection by contributing to a proinflammatory intrahepatic microenvironment resulting in accelerated liver fibrogenesis [43]. In a retrospective study of 21 patients with chronic HCV and type 2 diabetes mellitus treated with interferon-free DAA-based therapy, mean glucose significantly decreased during treatment (mean decrease −52.86 mg/dl, *p* = 0.007). In this study, hemoglobin A1C values were available for 10 patients, and were reduced in 8 (mean reduction −1.95 %, *p* = 0.021) [44]. Further, larger studies are needed to confirm the association between type 2 diabetes mellitus and chronic HCV, and the prioritization of these patients for HCV treatment.

##### Membranoproliferative glomerulonephritis

Chronic HCV is associated with multiple renal abnormalities, but membranoproliferative glomerulonephritis (MPGN) associated with cryoglobulinemia is the most common [45]. Non-cryoglobulinemic MPGN, IgA nephropathy, post-infectious glomerulonephritis, and focal and segmental glomerulosclerosis have been described in chronic HCV patients. Although cryoglobulins are observed in more than half of all chronic HCV patients, approximately 1 % of these patients develop mixed cryoglobulinemic vasculitis with end organ damage [46]. Patients with mixed cryoglobulinemic vasculitis usually manifest with mild to moderate renal insufficiency as well as microscopic proteinuria and hematuria. Renal biopsy of these patients usually shows classic MPGN with immune complex deposition in the glomeruli [47]. Therapy for HCV MPGN is oriented at the immune response and eradication of HCV. HCV therapy is usually associated with improvement or resolution of renal and dermatological manifestations of mixed cryoglobulinemic vasculitis [48]. The goal is to treat HCV with newer DAA agents that would result in improved renal function.

##### Neurological impairments

Involvement of the central nervous system in chronic hepatitis is an intense area of investigation. It is hypothesized that the effect of HCV on the brain is likely to be a direct infection with indirect stimulation of neurotoxic cytokines and development of CNS vasculitis. Approximately half of all HCV patients report some degree of impaired neurocognition [49, 50]. Imaging studies of the brain in patients with chronic HCV demonstrate vascular changes that could explain some of the defects in cognitive function observed [51]. Whether HCV actively replicates in the brain is an area of intense debate. Studies have demonstrated HCV RNA and negative strand RNA in brain tissue and cerebrospinal fluid suggesting active HCV replication in the CNS [52, 53]. Similarly, HCV proteins have also been detected in astrocytes by immunohistochemistry, although this is not necessarily representative of direct infection [54]. Indirect activation of monocytes by engaging toll-like receptor 2 on dendritic cells has also been suggested as a potential mechanism for HCV neurotoxicity [55]. Recently, post mortem studies of brain tissue of patients with HIV and HCV indicate a strong independent association of HCV and not HIV to small-vessel cerebrovascular disease [56].

Advanced liver disease by itself can affect neurocognitive function independent of HCV status. These patients develop porto-venous shunts that transport neurotoxic products directly to the systemic circulation. One hypothesis for the neurocognitive impairment associated with advanced liver disease is that neurotoxins such as ammonia may then cross the blood brain barrier [11]. Increased permeability of the blood brain barrier to ammonia [57] and increased brain uptake and utilization of ammonia among patients with hepatic encephalopathy compared to those without has been demonstrated [58]. More studies are required to clarify an independent association of HCV infection on neurological impairment independent of that occurring as a result of underlying liver fibrosis. HCV has also been associated with peripheral neuropathy with both sensory and motor impairment. It is likely that mixed cryoglobulinemic vasculitis may explain this phenomenon in some patients, while many without cryoglobulinemic vasculitis also have peripheral neurological symptoms.

##### Sicca syndrome

Sicca syndrome occurs in most patients with Sjogrens’ syndrome. Several host factors contribute to the development of Sjogren’s syndrome and chronic HCV has also been implicated as one of the causative factors [59]. It is unclear if HCV is truly a causative agent of, or whether patients with HCV present with clinical symptoms that mimic sicca syndrome. Although histologically similar, HCV associated sicca syndrome presents at a later age in men along with elevated serum cryoglobulins and rheumatoid factor, low serum complement, positive antinuclear antibody and negative Ro/La [60]. Treatment of chronic HCV results in resolution of symptoms in patients with sicca syndrome [61].

##### Porphyria cutanea tarda

Porphyria cutanea tarda (PCT) is the most common of the porphyrias and is associated with chronic HCV infection [62]. The biochemical abnormality that manifests as PCT is the decreased activity of the enzyme uroporphyrinogen decarboxylase [63]. Associated clinical conditions such as iron overload, alcohol abuse, and estrogens accentuate the symptoms of PCT [62]. Almost half of all PCT patients have chronic HCV infection, while PCT occurs in 1–5 % of all patients with chronic HCV [64]. We still do not completely understand the underlying mechanisms involved in this association. In general, treatment for HCV is associated with normalized enzymatic activity, ALT levels, urine porphyrins and skin lesions [65]. It is unclear if the new emergent DAA therapy will have an impact on this peculiar biochemical abnormality observed in HCV patients.

##### Lichen planus

Lichen planus is a chronic mucocutaneous disease of inflammatory origin. The common areas involved include limbs, face, scalp, nails and mucosal surfaces in gastrointestinal and genitourinary tracts [66]. Approximately 10–23 % of patients with chronic HCV manifest lichen planus [67]. It is likely that the underlying mechanism for the higher prevalence of lichen planus is immune related. Recent studies have reported shared peptide homology between HCV and desmoglein-3 as a potential antigenic mimicry mechanism for lichen planus [68]. Treatment of HCV with interferon-based therapy has not resulted in a resolution of lichen planus in most studies [69, 70], leaving providers without an optimal treatment regime for the management of patients with HCV and lichen planus.

#### Manifestations possibly associated with chronic HCV

These include several clinical syndromes that have been reported to occur at a higher frequency in patients with HCV. The clinical data that support each situation are primarily from case reports or case series rather than controlled studies. Hence, it is unclear to establish a direct causative role for HCV and these clinical syndromes.

##### Thyroid abnormalities

Thyroid abnormalities are more commonly reported with HCV infected patients in relation to interferon-alfa treatment. Approximately 15 % of all patients receiving interferon-alfa based HCV therapy will report clinical thyroid dysfunction. Interestingly, more than 35 % of these patients also develop thyroid autoantibodies. The mechanism for these includes an autoimmune phenomenon induced by immune-based therapy, which are often persistent even after discontinuation of therapy. Several studies have also associated the presence of abnormal thyroid autoantibodies in approximately one-tenth of all patients with chronic HCV prior to initiation of therapy [71]. Some investigators have detected HCV RNA in thyroid tissues, while others have productively infected thyroid cell lines with HCV. The authors hypothesize that HCV infection of thyrocytes could result in a localized inflammatory response and initiate autoimmune injury [72, 73]. All HCV patients should be screened for thyroid dysfunction and treated appropriately. Some clinicians also monitor those with abnormal autoantibodies closely for development of thyroiditis and symptoms. It will be interesting to see if eradication of HCV without the use of interferon-alfa using new DAA agents should ameliorate thyroid dysfunction in patients with HCV.

##### Autoantibodies

Circulating autoantibodies are high in patients with chronic HCV when compared to normal individuals. Mostly, these include cryoglobulins (60 %), rheumatoid factor (60 %), antinuclear antibody (40 %), antithyroid (35 %), anticardiolipin (15 %), and antismooth muscle antibodies (7 %) [3]. Alarmingly, half of all patients with chronic HCV present with at least one immunological abnormality [3]. None of these autoantibodies are associated with clinical abnormalities except for mixed cryoglobulins.

##### Mooren’s corneal ulcers

Several studies have associated rare ocular syndromes such as Mooren’s ulcer with HCV infection [74]. HCV infection is associated with several ocular conditions including keratitis, sicca syndrome likely from reduced lacrimation. Mooren’s ulcer is a rare, painful peripheral corneal ulceration usually manifesting without any associated scleritis [74]. The pathogenesis of this phenomenon in HCV patients is not completely understood. HCV infection is also associated with episcleritis, retinal vasculitis and retinopathy and increased intra ocular pressures compared to the general population [75, 76].

##### Cardiovascular events

Chronic HCV is associated with accelerated atherogenesis [77]. A systematic review found that the risk of developing early carotid artery atherosclerosis (measured by intima-media thickness) and the risk of advanced atherosclerosis (measured by the detection of carotid artery plaques) were about four times higher among patients with HCV compared to non-infected patients [78]. Several HCV cohorts have also reported an increase in cardiovascular disease related mortality [79], including a large cohort of over 10,000 HCV antibody positive blood donors, among whom there was a 2 fold increase in cardiovascular mortality (HR = 2.21, 95 % CI: 1.41, 3.46) compared to HCV antibody negative donors [80]. It is unclear why there is an increased risk of vascular events in patients with chronic HCV, but it is plausible that underlying proinflammatory cytokines present at high levels in patients could contribute to a proatherogenic milieu. Interestingly, a recent study reported greater endothelial dysfunction and increased carotid intima medial thickness among patients with genotype 1 infection compared with non-genotype 1 infection suggesting genotypic differences in vascular risk [81]. Furthermore, since the presence of diabetes mellitus is also increased among patients with chronic HCV, this could contribute as a significant co-factor in accelerating cardiac events. As the HCV-infected population ages, the risk of cardiac events also increases considerably. In a recent large study, treatment of HCV with interferon and ribavirin was associated with a 23 and 38 % reduction in acute coronary syndrome and ischemic stroke over an 8-year study period [8]. The recent development of DAA therapy would permit expanded treatment of patients to reduce the cardiovascular morbidity and mortality.

## Conclusions

Chronic HCV is associated with multi-system manifestations beyond the liver. In general, the occurrence of these manifestations is unpredictable and occurs irrespective of the stage of the liver disease. Recent developments in HCV therapeutics have established simpler, all oral, highly effective DAA agents as first line of treatment [82]. The high cost of DAA therapy has resulted in treatment being reserved for patients with the most advanced liver fibrosis [83]. A careful examination for extrahepatic manifestations in chronic HCV patients is warranted to prioritize HCV therapy for these individuals, since eradication of HCV is often associated with cessation of extrahepatic manifestations. Expanding the treatment to all those who have extrahepatic symptoms could improve the morbidity and mortality associated with systemic HCV disease.
